# Sustained inflation and incremental mean airway pressure trial during conventional and high-frequency oscillatory ventilation in a large porcine model of acute respiratory distress syndrome

**DOI:** 10.1186/1471-2253-6-8

**Published:** 2006-06-22

**Authors:** Ralf M Muellenbach, Markus Kredel, Bernd Zollhoefer, Christian Wunder, Norbert Roewer, Joerg Brederlau

**Affiliations:** 1 Department of Anaesthesiology, University of Wuerzburg Oberduerrbacher Strasse 6, 97080 Wuerzburg, Germany

## Abstract

**Background:**

To compare the effect of a sustained inflation followed by an incremental mean airway pressure trial during conventional and high-frequency oscillatory ventilation on oxygenation and hemodynamics in a large porcine model of early acute respiratory distress syndrome.

**Methods:**

Severe lung injury (Ali) was induced in 18 healthy pigs (55.3 ± 3.9 kg, mean ± SD) by repeated saline lung lavage until PaO_2 _decreased to less than 60 mmHg. After a stabilisation period of 60 minutes, the animals were randomly assigned to two groups: Group 1 (Pressure controlled ventilation; PCV): FIO_2 _= 1.0, PEEP = 5 cmH_2_O, V_T _= 6 ml/kg, respiratory rate = 30/min, I:E = 1:1; group 2 (High-frequency oscillatory ventilation; HFOV): FIO_2 _= 1.0, Bias flow = 30 l/min, Amplitude = 60 cmH_2_O, Frequency = 6 Hz, I:E = 1:1. A sustained inflation (SI; 50 cmH_2_O for 60s) followed by an incremental mean airway pressure (mPaw) trial (steps of 3 cmH_2_O every 15 minutes) were performed in both groups until PaO_2 _no longer increased. This was regarded as full lung inflation. The mPaw was decreased by 3 cmH_2_O and the animals reached the end of the study protocol. Gas exchange and hemodynamic data were collected at each step.

**Results:**

The SI led to a significant improvement of the PaO_2_/FiO_2_-Index (HFOV: 200 ± 100 vs. PCV: 58 ± 15 and T_Ali_: 57 ± 12; p < 0.001) and PaCO_2_-reduction (HFOV: 42 ± 5 vs. PCV: 62 ± 13 and T_Ali_: 55 ± 9; p < 0.001) during HFOV compared to lung injury and PCV. Augmentation of mPaw improved gas exchange and pulmonary shunt fraction in both groups, but at a significant lower mPaw in the HFOV treated animals. Cardiac output was continuously deteriorating during the recruitment manoeuvre in both study groups (HFOV: T_Ali_: 6.1 ± 1 vs. T_75_: 3.4 ± 0.4; PCV: T_Ali_: 6.7 ± 2.4 vs. T_75_: 4 ± 0.5; p < 0.001).

**Conclusion:**

A sustained inflation followed by an incremental mean airway pressure trial in HFOV improved oxygenation at a lower mPaw than during conventional lung protective ventilation. HFOV but not PCV resulted in normocapnia, suggesting that during HFOV there are alternatives to tidal ventilation to achieve CO_2_-elimination in an "open lung" approach.

## Background

Current treatment goals in patients with acute respiratory distress syndrome (ARDS) are to maintain adequate oxygenation while minimizing ventilator-associated lung injury (VILI) [1,2]. Several experimental studies have shown that mechanical ventilation (MV) can cause or perpetuate lung injury if alveolar overdistension, repetitive collapse and reopening of alveolar units occur [3,4]. Based on these findings, it is now recommended that patients with ARDS should be ventilated with low tidal volumes to limit end-inspiratory pressure and alveolar overdistension, while maintaining the lung open with sufficient positive end-exspiratory pressure (PEEP) to prevent alveolar collapse [5,6]. Although this lung protective strategy significantly reduces mortality in patients with ARDS, it is recognized that low peak pressure ventilation favours further lung derecruitment [7,8]. Recruitment manoeuvres (RM) composed of periodic sighs or sustained inflations to reexpand the collapsed lung units have been proposed as an adjunct to MV during general anaesthesia and ARDS [9-11]. Such manoeuvres may lead to immediate improvements in respiratory compliance and oxygenation. However, in clinical practice, many patients with ARDS remain hypoxemic using conventional lung protective ventilatory approaches, and the morbidity and mortality of patients suffering from ARDS remains unacceptably high [5,6,12].

High-frequency oscillatory ventilation (HFOV) is an alternative to conventional lung protective ventilation. The application of tidal volumes less than 6 ml/kg body weight combined with a high mean airway pressure (mPaw) to avoid lung overdistension and derecruitment, provides a basis for an alternative lung protective ventilatory strategy [13,14]. In a multicenter randomized controlled trial, Derdak and colleagues showed a significant early improvement of the PaO_2_/FiO_2_-Ratio in the HFOV group, while applying a significantly higher mPaw [15]. In small animal studies of acute lung injury and neonatal and paediatric respiratory distress syndrome, HFOV, utilizing an aggressive lung recruiting strategy, was shown to be safe and improved oxygenation compared to conventional mechanical ventilation [16-18]. HFOV, as a rescue therapy, is increasingly employed in intensive care patients with severe ARDS who remain hypoxemic during conventional ventilation.

The aim of this study was to evaluate the immediate effect of a sustained inflation (SI = 50 cmH_2_O) followed by an incremental and matched mean positive airway pressure augmentation in a large porcine model of severe ARDS with two different lung protective ventilation strategies (low-tidal pressure controlled ventilation versus HFOV) on gas exchange and hemodynamics. We hypothesized, that HFOV will reverse hypoxia at a lower mPaw than conventional lung protective ventilation, thereby aiming for a more lung protective recruitment strategy.

## Methods

The study was approved by the Laboratory Animal Care and Use Committee of the District of Unterfranken, Germany and conducted in compliance with the National Institutes of Health guidelines for ethical animal research.

### Animal preparation

Eighteen healthy female pigs (weight 55.3 ± 3.9 kg, mean ± SD), Pietrain breed, were fasted for 24 hours with free water access. After intramuscular premedication with ketamin (10 mg/kg), xylazine hydrochloride (1 mg/kg) and atropine (25 μg/kg) an intravenous line was obtained, and anaesthesia was induced with 10 mg/kg thiopental. All animals were positioned supine and orally intubated with a cuffed 8.5-mm ID endotracheal tube with an additional side lumen (Rueschelit^®^, Ruesch, Kernen, Germany). Anaesthesia and muscle relaxation were maintained with continuous infusion of 5–10 mg/kg/h thiopental, 0.01 mg/kg/h fentanyl and 0.1 mg/kg/h pancuronium throughout the experiment. Pressure controlled ventilation (PCV) was adjusted with a PEEP of 5 cmH_2_O, a tidal volume of 6 ml/kg, a respiratory rate (RR) of 30/min and an inspiratory to exspiratory ratio (I:E) of 1:1. The inspiratory oxygen fraction (FIO_2_) was kept at 1.0. Continuous electrocardiography (Servomed^®^, Hellige, Freiburg i. Br., Germany), pulsoxymetry, capnography and distal tracheal pressure monitoring were performed. A 20-gauge arterial catheter (Vygon, Ecouen, France) and a 9.0-French introducer sheath (Arrow, Reading, PA, USA) were inserted in the left carotid artery and in the right internal jugular vein respectively by using real-time ultrasound guidance (SonoSite 180 Plus^®^, SonoSite Inc., Botell, WA, USA). A 7.5-French flow-directed pulmonary artery catheter (831F75, Edwards Lifescience, Irvine, CA, USA) was inserted under transduced pressure guidance. The core temperature, as determined by the pulmonary artery catheter, was maintained at 38.0° ± 0.5°C during the experiment by using a heating pad. After a bolus of 500 ml colloid solution (Voluven 6% HES 130/0.4, Fresenius Kabi, Bad Homburg, Germany) prior the recruitment manoeuvre a continuous infusion of 4–5 ml/kg/h balanced electrolyte solution was administered for adequate hydration.

### Data acquisition

All hemodynamic data were taken in the supine position referenced to atmospheric pressure at the mid-chest level. Mean arterial pressure (MAP), mean pulmonary artery pressure (MPAP), central venous pressure (CVP) and pulmonary artery occlusion pressure (PCWP) were recorded (Servomed^®^, Hellige, Freiburg i. Br., Germany). Heart rate (HR) was traced by the electrocardiogram. Cardiac output (CO) was measured by using standard thermodilution techniques and expressed as the mean of three consecutive measurements (Explorer^®^, Edwards Lifescience, Irvine, CA, USA), each consisting of a 10 ml bolus of ice-cold saline into the right atrium randomly during the respiratory cycles. Blood samples were drawn from systemic arterial and pulmonary arterial lines and were immediately analyzed for oxygen pressure (PO_2_), carbon dioxide pressure (PCO_2_) and pH using standard blood gas electrodes (ABL 505^®^, Radiometer, Bronshoj, Denmark). In each sample, hemoglobin and oxygen saturation (SO_2_) were measured using specific spectroscopy (OSM3^®^, Radiometer, Copenhagen, Denmark). Arterial (CaO_2_), mixed venous (CvO_2_) and pulmonary capillary (CCO_2_) oxygen contents (ml/dl) were calculated using the standard formula: Oxygen content = hemoglobin concentration × 1.34 × (% O_2 _saturation/100) + PO_2 _× 0.0031. Oxygen delivery (DO_2_) and consumption (VO_2_) were determined using the conventional formulas DO_2 _= CO × CaO_2 _× 10 and VO_2 _= CO × (CaO_2 _- CvO_2_) × 10. The standard shunt equation was used to calculate the amount of venous admixture (Qs/Qt): (CCO_2 _- CaO_2_)/(CCO_2 _- CvO_2_). Mean airway pressure was measured at the side lumen ending at the tubes tip using an air filled pressure transducer (PM8050^®^, Draeger, Luebeck, Germany) referenced to atmospheric pressure. The oxygenation index was calculated using the following formula: OI = (FiO2 × mPaw × 100)/PaO_2_[19].

### Experimental protocol

After instrumentation the animals were stabilized for 30 min in the supine position and baseline measurements (T_baseline_) were performed. Severe lung injury was induced by bilateral pulmonary lavages with 30 ml/kg isotonic saline (38°C), repeated every 10 minutes until PaO_2 _decreased to less than 60 mmHg and remained stable for 60 minutes with unchanged ventilatory parameters.

### Recruitment manoeuvres and ventilator adjustment

After stabilisation post injury measurements (T_ali_) were obtained and the animals were randomly assigned to the following treatment groups (n = 9/group):

1. PCV: FIO_2 _= 1.0, PEEP = 5 cmH_2_O, V_T _= 6 ml/kg, RR = 30/min, I:E = 1:1

2. High-frequency oscillatory ventilation (HFOV): FIO_2 _= 1.0, Bias flow = 30 l/min, Amplitude = 60 cmH_2_O, Frequency = 6 Hz, I:E = 1:1 (Sensor Medics 3100 B, Yorba Linda, CA, USA)

**Figure 1:**
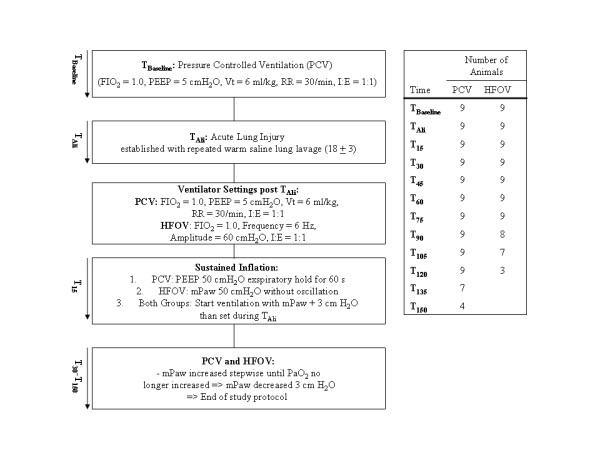
**Study protocol and time course**. FiO_2 _= fraction of inspired oxygen; PEEP = positive endexspiratory pressure; Vt = tidal volume; RR = respiratory rate; I:E = inspiratory:exspiratory-ratio; PCV = pressure controlled ventilation; HFOV = High-frequency oscillatory ventilation; mPaw = mean pulmonary airway pressure; PaO_2 _= arterial oxygen pressure

A sustained inflation (SI) was applied by an exspiratory hold for 60 s (PEEP 50 cmH_2_O) in the PCV group and increasing the mean airway pressure (mPaw) to 50 cmH_2_O without oscillation for 60 s in the HFOV group. In both groups, the mPaw was then adjusted 3 cmH_2_O higher than after acute lung injury (T_Ali_) and increased in steps of 3 cmH_2_O until PaO_2 _no longer increased or began to decline. This was regarded as full lung inflation or the beginning of lung overdistension. Afterwards mPaw was reduced by 3 cmH_2_O and the animals reached the end of the study protocol [20]. A 15-min equilibration period between each modification was followed by measurements of hemodynamics, arterial blood gases and respiratory parameters. Intrinsic PEEP was measured during PCV by means of an endexspiratory occlusion manoeuvre (5 secs) at each time point and was always less than 1 cmH_2_O. Pulmonary artery blood gases were measured at following time points: T_Baseline_, T_Ali_, T_60 _and T_120_. At the end of the study the animals were killed using an intravenous overdose of sodium thiopental and T61^® ^(Intervet, Unterschleissheim, Germany). The study protocol and time course of the whole experiment is outlined in Fig. 1.

### Statistical analysis

The data were tested for normal distribution using the Kolmogorov-Smirnov test. Values are reported as mean ± SD. The data were analyzed using SigmaStat for Windows, version 2.03 (Systat Software Inc., Point Richmond, USA). Two-way analysis of variance (ANOVA) for repeated measurements was used for data analysis. Student-Newman-Keuls post hoc test was used for comparison of significant ANOVA results within and between the groups. P values of less than 0.05 were considered significant. Because of different numbers of animals during the time points T_90 _to T_150 _no data were statistically analyzed.

## Results

**Figure 2:**
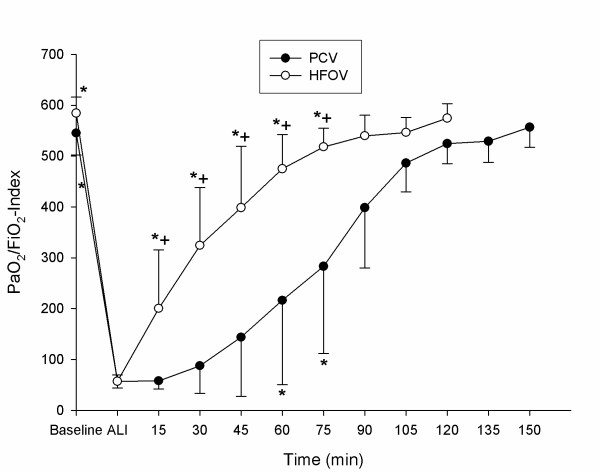
**PaO_2_/FiO_2 _– Index**. PaO_2_/FiO_2 _– Index (mean ± SD) during recruitment manoeuvre. PCV = pressure controlled ventilation; HFOV = High-frequency oscillatory ventilation. * p < 0.001 vs. T_Ali_; + p < 0.001 HFOV vs. PCV. (n = 9 until 75 min).

**Figure 3:**
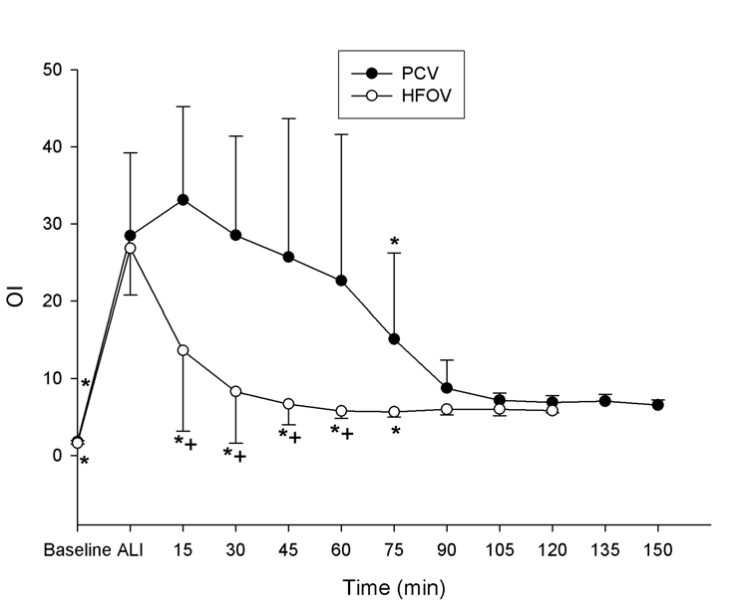
**Oxygenation Index (OI)**. Oxygenation Index (mean ± SD) during recruitment manoeuvre. PCV = pressure controlled ventilation; HFOV = High-frequency oscillatory ventilation. * p < 0.001 vs. T_Ali_; + p < 0.001 HFOV vs. PCV. (n = 9 until 75 min).

**Figure 4:**
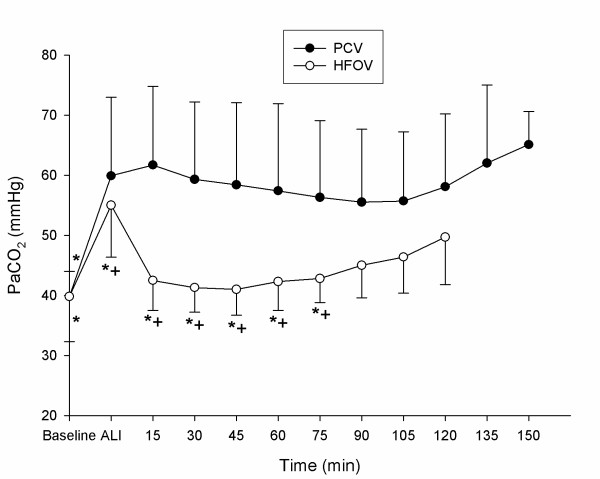
**Partial pressure of arterial carbon dioxide(PaCO_2_)**. PaCO_2 _(mean ± SD) during recruitment manoeuvre. PCV = pressure controlled ventilation; HFOV = High-frequency oscillatory ventilation. * p < 0.01 vs. T_Ali_; + p < 0.01 HFOV vs. PCV. (n = 9 until 75 min).

**Figure 5:**
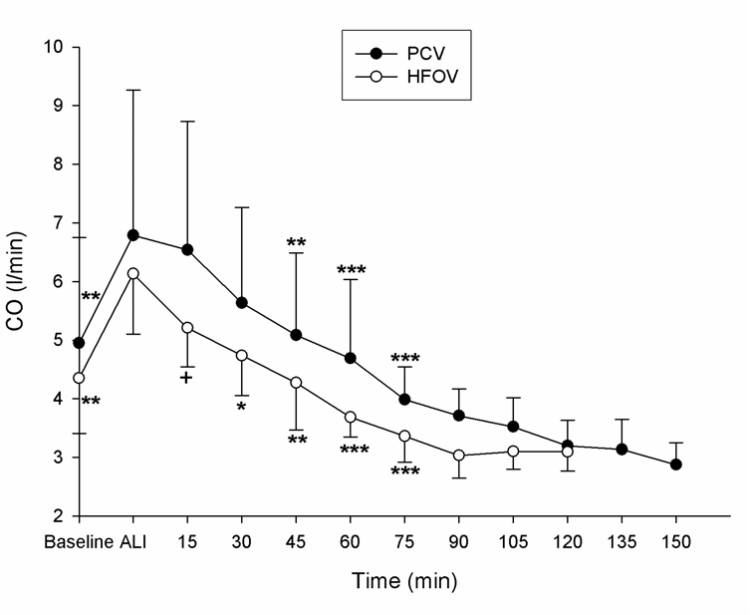
**Cardiac output (CO)**. CO (mean ± SD) during recruitment manoeuvre. PCV = pressure controlled ventilation; HFOV = High-frequency oscillatory ventilation. * p < 0.05, ** p < 0.01 and *** p < 0.001 vs. T_Ali_; + p < 0.01 HFOV vs. PCV. (n = 9 until 75 min).

Detailed data regarding hemodynamics, gas exchange and respiratory parameters are presented in tables 1 and 2. PaO_2_-, OI-, PaCO_2 _and CO-changes during the experimental period are displayed in figures 2, 3, 4, 5.

**Table 1:** Hemodynamic variables during recruitment manoeuvre.

	**Group**	**T **_baseline_	**T**_ALI_	**T**_15_	**T**_30_	**T**_45_	**T**_60_
**Number of**	PCV	9	9	9	9	9	9
**animals**	HFOV	9	9	9	9	9	9
**HR**	PCV	71 ± 18	85 ± 22	85 ± 18	76 ± 20	73 ± 24	73 ± 23
**[/min]**	HFOV	66 ± 9	79 ± 6	67 ± 5*+	63 ± 3*	62 ± 4*	64 ± 5*
**MAP**	PCV	75 ± 13***	93 ± 11	84 ± 17	86 ± 11	86 ± 10	86 ± 10
**[mmHg]**	HFOV	71 ± 8***	94 ± 9	87 ± 7	87 ± 5	85 ± 6	83 ± 7**
**CVP**	PCV	4.9 ± 2.4***	7.1 ± 2.3	7.6 ± 1.8	8.1 ± 1.8	8.6 ± 2.1**	9.4 ± 2.5***
**[mmHg]**	HFOV	5.2 ± 2.2**	6.4 ± 2.5	9.7 ± 2.0***+	10.4 ± 2.1***+	10.7± 1.9***+	11.9 ± 2.0***+
**MPAP**	PCV	19 ± 5***	37 ± 6	34 ± 5***	31 ± 4***	28 ± 4***	30 ± 3***
**[mmHg]**	HFOV	17 ± 2***	34 ± 5	30 ± 6**+	27 ± 4***	29 ± 4***	31 ± 4**
**PCWP**	PCV	7.3 ± 2.9	8.7 ± 2.1	10.2 ± 2.0	10.0 ± 2.3	12.0 ± 3.0***	12.6 ± 3.0***
**[mmHg]**	HFOV	7.4 ± 1.6**	9.7 ± 3.7	12.2 ± 2.8**	13.0 ± 2.9***+	12.8 ± 1.8***	14.0 ± 1.9***
**Qs/Qt**	PCV	0.09 ± 0.05***	0.54 ± 0.10				0.31 ± 0.17 **
**(ratio)**	HFOV	0.06 ± 0.01***	0.52 ± 0.07				0.10 ± 0.04***+

	**Group**	**T**_75_	**T**_90_	**T**_105_	**T**_120_	**T**_135_	**T**_150_

**Number of**	PCV	9	9	9	9	7	4
**animals**	HFOV	9	8	7	3		
**HR**	PCV	71 ± 18	74 ± 18	80 ± 18	83 ± 19	92 ± 18	100 ± 16
**[/min]**	HFOV	67 ± 8	77 ± 10	82 ± 13	97 ± 13		
**MAP**	PCV	83 ± 7*	79 ± 6	76 ± 10	75 ± 8	73 ± 9	77 ± 4
**[mmHg]**	HFOV	78 ± 6***	77 ± 8	80 ± 6	80 ± 5		
**CVP**	PCV	10.4 ± 2.4***	11.1 ± 2.0	11.7 ± 2.1	12.7 ± 3.0	13.4 ± 2.3	14.5 ± 2.5
**[mmHg]**	HFOV	12.8 ± 2.0***+	13.1 ± 1.8	13.4 ± 2.6	14.7 ± 2.9		
**MPAP**	PCV	32 ± 3***	33 ± 3	35 ± 4	37 ± 3	39 ± 5	41 ± 2
**[mmHg]**	HFOV	32 ± 4*	35 ± 5	36 ± 4	37 ± 4		
**PCWP**	PCV	13.4 ± 2.1***	13.6 ± 2.8	15.4 ± 3.1	17.3 ± 3.5	19.0 ± 2.7	19.3 ± 1.9
**[mmHg]**	HFOV	15.9 ± 2.4***+	16.4 ± 2.5	16.3 ± 3.3	18.0 ± 2.6		
**Qs/Qt**	PCV				0.06 ± 0.02		
**(ratio)**	HFOV				0.04 ± 0.02		

Data are mean ± standard deviation; Two-way-ANOVA with repeated measurements (Student-Newman-Keuls post hoc test): * p < 0.05 vs. T_Ali_; ** p < 0.01 vs. T_Ali_; *** p < 0.001 vs. T_Ali_; HFOV compared with PCV: + p < 0.01. PCV = pressure controlled ventilation; HFOV = High-frequency oscillatory ventilation; HR = heart rate; MAP = mean arterial pressure; CVP = central venous pressure; MPAP = mean pulmonary artery pressure; PCWP = pulmonary capillary wedge pressure; Qs/Qt = pulmonary shunt fraction.

**Table 2:** Variables of Gas exchange during recruitment manoeuvre.

	**Group**	**T **_baseline_	**T**_ALI_	**T**_15_	**T**_30_	**T**_45_	**T**_60_
**Number of**	PCV	9	9	9	9	9	9
**animals**	HFOV	9	9	9	9	9	9
**pHa**	PCV	7.45 ± 0.06***	7.32 ± 0.06	7.31 ± 0.06	7.33 ± 0.05	7.34 ± 0.06	7.34 ± 0.06
	HFOV	7.48 ± 0.09***	7.35 ± 0.04	7.43 ± 0.05*+	7.45± 0.06**+	7.45 ± 0.05**+	7.44 ± 0.05*+
**DO**_2_	PCV	794 ± 312	781 ± 216	740 ± 145	722 ± 133	677 ± 114	644 ± 110
**(ml/min/m**^2^**)**	HFOV	662 ± 157	724 ± 152	710 ± 105	677 ± 99	624 ± 117	547 ± 59*
**VO**_2_	PCV	184 ± 42*	221 ± 72				194 ± 44
**(ml/min/m**^2^**)**	HFOV	165 ± 37*	204 ± 52				188 ± 24
**SvO**_2_	PCV	83 ± 6***	60 ± 14				69 ± 10*
**[%]**	HFOV	84 ± 3***	62 ± 8				72 ± 5**
**mPaw**	PCV	10 ± 1***	15 ± 2	18 ± 2***	20 ± 2***	23 ± 2***	26 ± 2***
**[cmH2O]**	HFOV	9 ± 0***	15 ± 1	18 ± 1***	21 ± 1***	24 ± 1***	27 ± 1***
**PIP**	PCV	15 ± 1***	25 ± 3	27 ± 3***	28 ± 3***	30 ± 3***	33 ± 3***
**[cmH2O]**	HFOV	14 ± 1***	25 ± 2	20 ± 1***+	23 ± 1***+	26 ± 1***+	29 ± 1***+

	**Group**	**T**_75_	**T**_90_	**T**_105_	**T**_120_	**T**_135_	**T**_150_

**Number of**	PCV	9	9	9	9	7	4
**animals**	HFOV	9	8	7	3		
**pHa**	PCV	7.35 ± 0.06	7.35 ± 0.05	7.35 ± 0.05	7.33 ± 0.05	7.31 ± 0,06	7.30 ± 0,04
	HFOV	7.43 ± 0.04*+	7.41± 0.05	7.39 ± 0.04	7.38 ± 0.04		
**DO**_2_	PCV	591 ± 71*	571 ± 68	551 ± 65	506 ± 73	494 ± 89	475 ± 74
**(ml/min)**	HFOV	503 ± 71**	461 ± 72	478 ± 60	489 ± 92		
**VO**_2_	PCV				183 ± 27		
**(ml/min)**	HFOV				194 ± 18		
**SvO2**	PCV				70 ± 4		
**[%]**	HFOV				66 ± 9		
**mPaw**	PCV	28 ± 1***	31 ± 2	34 ± 1	36 ± 2	37 ± 4	36 ± 2
**[cmH2O]**	HFOV	29 ± 2***	32 ± 3	33 ± 4	33 ± 1		
**PIP**	PCV	35 ± 2***	38 ± 2	42 ± 3	43 ± 4	45 ± 6	45 ± 2
**[cmH2O]**	HFOV	31 ± 2***+	34 ± 3	35 ± 4	35 ± 1		

Data are mean ± standard deviation; Two-way-ANOVA with repeated measurements (Student-Newman-Keuls post hoc test): * p < 0.05, ** p < 0.01, *** p < 0.001 vs. T_Ali_; HFOV compared with PCV: + p < 0.001. PCV = pressure controlled ventilation; HFOV = High-frequency oscillatory ventilation; DO_2 _= oxygen delivery; VO_2 _= oxygen consumption; S_v_O_2 _= mixed venous oxygen saturation; mPaw = mean pulmonary airway pressure; PIP = peak pulmonary airway pressure.

### Lung injury

All animals survived the complete study period. Acute lung injury was induced in all animals by means of repeated saline lung lavages (18 ± 3) with significant changes (p ≤ 0.05) in pHa, PaCO_2_, OI, PaO_2_/FiO_2_-Index, VO_2_, SvO_2_, mPaw, PIP, MAP, CVP, MPAP, Qs/Qt – ratio and CO: pHa decreased from 7.46 ± 0.08 to 7.35 ± 0.06, PaCO_2 _increased from 39.8 ± 5.9 to 54.9 ± 11.8 mmHg, OI increased from 1.7 ± 0.2 to 27.6 ± 8.5, PaO_2_/FiO_2_-Index declined from 564 ± 41.9 to 57 ± 12.2 mmHg, VO_2 _increased from 174 ± 40.0 to 212 ± 61.5 ml/min./m^2^, SvO_2 _decreased from 83 ± 5.0 to 61 ± 11.0 %, mPaw increased from 9.5 ± 0.5 to 14.9 ± 1.3 cmH_2_O, PIP increased from 14.1 ± 1.2 to 24.8 ± 2.6 cmH_2_O, MAP increased from 73 ± 11 to 93 ± 10 mmHg, CVP increased from 5 ± 2.3 to 7 ± 2.4 mmHg, MPAP increased from 18 ± 3.8 to 36 ± 5.5 mmHg, Qs/Qt increased from 8 ± 4 to 53 ± 8 %, CO increased from 4.6 ± 1.4 to 6.4 ± 1.8 l/min. No significant differences could be detected between the 2 groups for the parameters tested at time points T_Baseline _and T_Ali _(tables 1 and 2, fig. 2, 3, 4 and 5).

### Pulmonary gas exchange

The SI and rising mPaw led to a significant improvement of OI and PaO_2_/FiO_2_-ratio in the HFOV group compared to T_Ali _and the PCV group from T_15 _to T_75 _(p ≤ 0.001 vs. T_Ali_and PCV; fig. 2 and 3). However, in the PCV group the SI was associated with a deterioration of OI and PaO_2_/FiO_2_-Ratio at T_15_. With rising mPaw oxygenation in the PCV group increased and was significantly improved at T_60 _and T_75 _(p ≤ 0.001 vs. T_Ali_; fig. 2 and 3). At T_60 _the SvO_2 _was significantly improved in both groups when compared to T_Ali _without detectable significant differences between the groups (p ≤ 0.05 vs. T_Ali_; table 2). PaCO_2 _improved significantly in the HFOV group compared to T_Ali _and the PCV group (p ≤ 0.001; fig. 4). The PCV animals remained hypercapnic with a PaCO_2 _greater 55 mmHg resulting in a pH of less than 7.35 throughout the experiment (fig. 4).

### Hemodynamics and oxygen delivery

HR and MAP showed a downward drift during the SI and incremental mPaw trial in both groups, but it was not necessary to stop the recruitment manoeuvre due to critical hemodynamic compromise.

HR was significantly lower at T_15 _in the HFOV vs. the PCV group and from T_15 _to T_60 _vs. T_Ali _(table 1). MAP significantly decreased at T_60 _in the HFOV animals and at T_75 _in both groups compared to T_Ali _(table 1). CVP and PCWP showed a significant elevation in the HFOV group from T_15 _to T_75 _compared to T_Ali _and CVP was significantly higher than in the PCV group during the same time course (table 1). MPAP was significantly lower in both groups compared to T_Ali _and at T_15 _significantly lower in the HFOV than PCV animals (table 1). CO was significantly lower and continuously falling in the HFOV group (fig. 5). At T_15 _CO was significantly lower in the HFOV compared to PCV group and from T_45 _to T_75 _CO was significantly decreased compared to T_Ali _in PCV animals (fig. 5). Qs/Qt was at T_60 _significant lower in both groups compared to injury and significant reduced in the HFOV versus PCV group (table 1).

## Discussion

This study investigated the impact of a sustained inflation followed by an incremental and matched mean airway pressure trial during HFOV and conventional lung protective ventilation on gas exchange and hemodynamics. The major findings of our study are: 1) Combination of HFOV and SI with rising mPaw improved oxygenation and resulted in a significant reduction of pulmonary shunt fraction at a lower mPaw than during conventional lung protective ventilation. 2) The SI and rising mPaw resulted in normocapnia in the HFOV but not in the PCV group 3) Both groups exhibited a continuously deterioration in cardiac output with rising mPaw.

The treatment of severe hypoxemia in ARDS is highly dependent on the recruitment and maintenance of lung volume [21,22]. This is related to the dynamic reopening of previously collapsed lung units by increasing transpulmonary pressure. In order to assess gas exchange at comparable levels, we chose to perform a SI followed by stepwise increases in mPaw, which was measured continuously in the trachea using a pressure transducer fast enough to detect pressure changes at 6 Hertz. In both groups we used the same SI of 50 cmH_2_O for 60 seconds and afterwards set the mPaw 3 cmH_2_O higher than after lung injury. The PaO_2_/FiO_2_-Ratio increased and the OI decreased significantly in the HFOV, but not in the PCV group (fig. 2 and 3). Our findings are similar to those of previous studies, showing that HFOV therapy is safe and results in early improvements in oxygenation compared to conventional ventilation. Since very small tidal volumes are generated with HFOV, there is little tidal recruitment of the injured lung, recruitment of lung volume appears to be essential in this setting [18,23]. The improvement in oxygenation seems to correspond with increases in lung volume [24]. In the HFOV animals this increase was presumably maintained after the SI resulting in significant PaO_2_/FiO_2 _enhancement. The favourable effect of the initial SI might have been reversed in the PCV group because the applied PEEP was possibly not high enough to maintain previously recruited lung areas open. Bond and co-workers failed to show acute volume recruitment after SI, even when PEEP was set above the critical closing pressure of the lung during large tidal volume ventilation [24]. In contrast, Foti et al. showed that volume recruitment manoeuvres can improve oxygenation and alveolar recruitment during conventional ventilation at low PEEP levels [9]. It has been suggested that the collapsed lung, in which low PEEP is being used, benefits more from a RM than lungs already ventilated with high PEEP [24,25]. In ARDS patients ventilated with low tidal volumes, derecruitment was reversed by a reexpansion manoeuvre or prevented by an adequate PEEP level [8].

In our study the stepwise rises in mPaw resulted in an improved oxygenation in both groups. At matched mPaw, HFOV resulted in a significant higher PaO_2_/FiO_2_-Ratio and a lower OI (fig. 2 and 3).

We did not try to achieve normocapnia. Ventilatory settings were fixed in both study groups. This resulted in hypercapnia in the conventional ventilation and in normocapnia in the HFOV group. This is presumably the result of a greater reduction in physiologic deadspace ratio in the HFOV animals, which was again most probably a result of a greater lung volume ensuring a better ventilation/perfusion relationship after the SI. These data are in contrast with recently published randomized controlled trial without RM, since normocapnia was not achievable with HFOV in human and experimental ARDS-trials [15,26].

### Hemodynamic effect

We detected changes in the hemodynamic status during the RM compared to T_Ali_. Because of the potential for hemodynamic compromise, we decided to apply just one SI and an incremental and not decremental mPaw trial. The higher mPaw applied during both respiratory modes was associated with an early and persistent decrease in CO, a persistent increase in CVP and PCWP and a small decrease in MAP. The reason for these changes was most likely the sustained increase in intrathoracic pressure as described in previous studies [15,27].

### Limitations

The major limitation of this study is that it was performed in pigs and not in patients. In our study we used a surfactant-depletion lung-lavage model of ARDS. This model does not represent the complexity of the lung injury in ARDS patients. In addition, it is known that surfactant depleted collapsed lungs respond better to PEEP application and RM than lungs with alveolar flooding [25,28].

We used PaO_2 _as a surrogate marker for lung volume recruitment, since lung volumes and pressure volume relationships have not been directly quantified [29]. It was shown recently, that generation of pressure volume curves is associated with further lung derecruitment [30]. Additional deterioration of oxygenation in our animals after lung injury would have implied a high risk of irreversible hypoxia.

Probably due to the severity of the experimentally induced ARDS, the compliance changes in our model were very consistent across the animals and linear throughout the incremental mPaw-trial. This linearity enabled us to increase mPaw in steps of 3 cmH_2_O in the PCV-group by PEEP-augmentation of 3 cm H_2_O. The results are not transferable to other animal models or humans with lungs characterized by different pressure-volume-relationships.

## Conclusion

In this saline lavage induced large porcine model of early severe ARDS, sustained inflations followed by an incremental mean airway pressure trial improved gas exchange and resulted in a significant reduction of pulmonary shunt fraction at a lower mPaw during HFOV than during conventional lung protective ventilation. Large animal long term trials with acquisition of histopathologic and inflammatory cytokine data are needed to evaluate whether the thereby possible reduction of mPaw results in further lung protection. Normocapnia was achieved in HFOV but not in the PCV group, suggesting that large volume swings might not be required to eliminate CO_2 _in an "open lung" approach.

## Competing interests

The author(s) declare that they have no competing interests.

## Authors' contributions

RMM conceived the study, collected and analysed the data and drafted the manuscript. MK and BZ collected data and performed the statistical analysis. CW and NR participated in the design of the study. JB designed the study protocol and helped writing the manuscript. All authors read and approved the final manuscript.

## Pre-publication history

The pre-publication history for this paper can be accessed here:


